# Innate Immunity and Phenoptosis

**DOI:** 10.1134/S0006297922120185

**Published:** 2023-01-13

**Authors:** Boris V. Chernyak, Konstantin G. Lyamzaev

**Affiliations:** 1 Belozersky Institute of Physico-Chemical Biology, Lomonosov Moscow State University, 119991 Moscow, Russia; 2 Russian Clinical Research Center for Gerontology, Pirogov Russian National Research Medical University, Ministry of Healthcare of the Russian Federation, 129226 Moscow, Russia

**Keywords:** pathogen-associated molecular patterns (PAMPs), damage-associated molecular patterns (DAMPs), mitochondrially-targeted antioxidants, inflammation, inflammasome, programmed death, phenoptosis

## Abstract

The hypothesis is proposed that activation of innate immunity is the primary mechanism of phenoptosis (programmed death of an organism). In support of the hypothesis, we discuss (i) the data on active release of signaling molecules from the cell producing excessive inflammation; (ii) the data on contribution of mitochondrial production of reactive oxygen species to immune response.

## INTRODUCTION

The concept of phenoptosis as an altruistic programmed death of individual organisms for the benefit of population and species was proposed by Vladimir Petrovich Skulachev more than two decades ago [1, 2]. V. P. Skulachev suggested that Darwinian selection could form the mechanisms of “cleansing a kinship community or a population from individual organisms that have become harmful to this community.” In particular, “septic shock… and stress-induced ischemic diseases of the brain and heart” were proposed to be considered as examples of phenoptosis [1]. At the same time, “slow phenoptosis” has been proposed as an equivalent term for programmed aging [1-3].

The concept of phenoptosis has gained considerable popularity and has served as a catalyst for numerous, both theoretical and experimental studies. In our opinion, there was an important gap in this concept, because no specific mechanism (or mechanisms) was proposed, which would be able to determine the fate of the individual organism in the course of phenoptosis. Such mechanisms have been suggested for a number of different types of programmed cell death including apoptosis, necroptosis, and pyroptosis. In this note, we will briefly present the hypothesis that activation of innate immune system serves as a primary mechanism for phenoptosis execution. A more detailed analysis of the literature in this area was published by us earlier [4].

## INNATE IMMUNITY AND PHENOPTOSIS

It is well known that activation of innate immunity in response to viral (including COVID-19) and bacterial infections, as well as to massive trauma (in particular, during surgical interventions), ischemic and toxic lesions often lead to severe illness and death. It is traditionally believed that this is the result of erroneous hyperstimulation of protective reactions, which serves as an evolutionary trade-off for the high efficiency of the immune system. Based on numerous observations, we suggested that such dangerous properties of the innate immunity are the result of an altruistic suicidal strategy fixed in evolution to protect population from the spread of epidemics and dangerous pathologies.

Convincing evidence in favor of our hypothesis follows from consideration of the basic principles of innate immunity. These principles, formulated by C. A. Janeway [5], state that pathogens in the organism are recognized by a relatively small number of receptors that are tuned not to the individual characteristics of the pathogen (as in the case of adaptive immunity), but to common features inherent in the large groups of pathogens, the so-called pathogen-associated molecular patterns (PAMPs). These patterns include bacterial wall lipopolysaccharides, some proteins and peptides, and viral and bacterial nucleic acids. The main property of PAMPs is their absence in the host organism. It soon became clear, however, that the same receptors recognize many molecular components of the host cells. They are called damage-associated molecular patterns (DAMPs). Initially, it was assumed that these molecules enter extracellular environment exclusively from the damaged cells, however, data gradually began to accumulate that many DAMPs are actively released by living cells. For example, mitochondria are an important source of DAMPs. This is partly due to their origin from endosymbiotic bacteria, although some mitochondrial DAMPs do not have bacterial homologues. Mitochondria in the cell produce ATP due to the energy of respiration and inevitably produce reactive oxygen species (ROS). Release of the damaged organelles from the cell is one of the mechanisms by which the cell is freed from the ROS-damaged mitochondria [6]. The mechanisms of this active process are not entirely clear. It is possible that some of the mitochondrial DAMPs are released into the extracellular environment as waste, however, it has been shown that inflammatory activation of immune cells significantly enhances the release of mitochondria, creating an amplification loop of inflammation. Another example of active release of DAMPs is secretion of vesicles by granulocytes. These immune cells release secretory granules loaded with antimicrobial peptides and lytic enzymes to defend against pathogens, however, many of these molecules are recognized as DAMPs. ATP, uric acid, and succinate are important low molecular weight metabolites involved in activation of inflammation after leaving the cell. Active ejection systems are known for all these molecules.

The most striking example of the active release of DAMPs are nuclear proteins HMGB1 and CIRP. Normally, they are involved in regulation of replication and transcription, but once outside the cell, they serve as powerful activators of immune response. Active release of nuclear DAMPs requires their post-translational modification, which allows them to enter cytoplasm, and then the release occurs due to exocytosis of secretory lysosomes [7, 8]. Apparently, this is due to active participation of the innate immunity reactions in the repair of tissue damage. At the same time, these and other DAMPs have been shown to be critical for the development of many pathologies. In particular, mice with the CIRP gene knockout survive sepsis, which causes death of the control animals [8]. Introduction of antibodies that intercept some DAMPs or inhibitors that block their receptors prevents the development of sepsis, aseptic systemic inflammation, ischemic lesions, etc. [9]. HMGB1 and CIRP inhibitors are considered as promising therapeutic agents for various inflammatory pathologies. These observations are very difficult to explain in terms of the protective function of immune system, but they are in good agreement with the hypothesis of participation of immune reactions in phenoptosis.

Research on the function of mitochondria in innate immunity is another crucial field that provides findings supporting our hypothesis. For the two main types of innate immunity cells, neutrophils and macrophages, very important role of mitochondrial ROS (mitoROS) production in inflammatory activation has been shown. In neutrophils, mitoROS stimulate assembly and activation of NADPH oxidases on the plasma membrane, which leads to the massive release of radicals (“oxidative burst”) [10]. In addition, mitoROS promote the release of various types of vesicles filled with lytic enzymes from neutrophils (the so-called degranulation). Finally, some stimuli cause the release of nuclear chromatin from neutrophils, which decondenses and forms extracellular traps (neutrophil extracellular traps, NETs), accompanied by necrotic cell death, NETosis. MitoROS generation is a prerequisite for NETosis, at least for some types of stimulation [11]. In macrophages, mitoROS also serve as an important signal for inflammatory cell activation [12].

Along with the immune cells, endothelial cells play an important role in the functioning of innate immune system. In response to inflammatory stimuli, they produce inflammatory cytokines and also express adhesion molecules on the surface, which are necessary for penetration of neutrophils from blood into the inflammatory foci. Our studies have shown that mitoROS are critically important for endothelial inflammatory activation mediated by the transcription factor NF-kB [13]. In both immune cells and endothelium, an important component of the inflammatory response is activation of the NLRP3 inflammasome, a large multiprotein complex that catalyzes maturation of the important inflammatory cytokines. It has been established that inflammasomes are localized on the surface of mitochondria and are activated with participation of mitoROS [14].

The mitochondria-targeted antioxidants (MTA) were primarily used to collect data on the function of mitoROS in innate immunity. These compounds selectively accumulate in the negatively charged mitochondrial matrix due to cationic residue and neutralize the action of mitoROS. These antioxidants effectively block inflammatory responses of both immune cells and endothelium. Over the past decade, a large amount of data has been accumulated on the therapeutic effect of MTAs in animal models of various pathologies. SkQ1, a mitochondrial-targeted antioxidant developed by V. P. Skulachev and co-workers, has been studied the most [15]. It has been shown that SkQ1 rescues mice in the model of acute aseptic inflammation [16], as well as in the model of pyelonephritis, where acute inflammation was caused by administration of bacterial preparations [17]. In addition, SkQ1 had a therapeutic effect in the models of ischemic stroke and myocardial infarction, as well as in the models of eye diseases [15]. All these pathologies largely depend on inflammatory processes, and this suggests that the anti-inflammatory effect of SkQ1, at least in part, determines its therapeutic efficacy. These data show that normal cellular functions (in particular, generation of mitoROS) are involved in formation of immune responses that could cause death of an organism (phenoptosis). It should be noted that, both in experimental and clinical studies, the long-term intake of traditional (usually natural) antioxidants did not show either a significant therapeutic effect or increase in life expectancy [18]. It can be assumed that there are two main reasons for this: (i) low effectiveness of natural antioxidants in acceptable doses, and (ii) existence of numerous “side effects” due to the fact that ROS are necessary for normal physiological processes. The evidence suggests that the mitochondria-targeted antioxidants can overcome these drawbacks, which is encouraging. The studies cited above showed that SkQ1 had a pronounced therapeutic effect in the models of acute inflammatory pathologies. The long-term use was not accompanied by the development of any pathologies in mice [19]. SkQ1 significantly increased average lifespan of the laboratory mice [20], and this effect was especially pronounced in the short-lived mutant line [21]. It could be assumed that such effect of SkQ1, at least in part, is associated with the suppression of innate immunity.

V. P. Skulachev’s concept of phenoptosis includes the idea of “slow phenoptosis”, which is equivalent to programmed aging. The hypothesis on the role of innate immunity in phenoptosis can be extended to this case. This has much in common with the hypothesis of “inflammatory aging” proposed by K. Franceschi [22]. In full agreement with this hypothesis, the long-term experiments on the selection of long-lived Drosophila flies led to the selection of flies with suppressed immune system [23]. A similar experiment, apparently, was set in nature during the evolution of bats. In these animals, due to a single gene point mutation, antiviral immunity is significantly reduced [24]. Bats have taken the path of peaceful coexistence with many viruses, which has made them a reservoir of extremely dangerous pathogens. Perhaps it is due to the weakened immunity that bats live much longer (10-20 years, and some species up to 40 years) than most animals of a similar size. It is assumed that the decrease in immunity in this case is compensated by very high body temperature that occurs during flight. It is possible that a similar strategy is also implemented in some birds (albatrosses, large parrots), which live much longer (100 years or more) than their flightless relatives.

The recently discovered features of the immune system in the long-lived rodents such as naked mole rat (*Heterocephalus glaber*) and blind mole rat (*Spalax* spp.) merit separate discussion. The naked mole rat has a higher ratio of myeloid and lymphoid cells compared to mice, enhanced production of pro-inflammatory cytokines in macrophages, and special subpopulation of neutrophils that actively produce antimicrobial peptides, which indicates high activity of the innate immunity [25]. At the same time, their RIPK3 and MLKL genes carry mutations, which are required for necroptosis, a type of necrotic cell death that causes severe inflammation. This correlates with the reduced infiltration of immune cells into the damaged areas of the skin [26]. Interestingly, the same phenotype is observed upon inhibition of the RIPK3 kinase activity or upon knockout of the corresponding gene in mice [26]. In addition, the naked mole rat lacks natural killer cells (NK cells) important for innate immunity, which are capable of destroying the virus-infected cells [25]. Adaptive immunity of the naked mole rat also has its own characteristics. It has been shown that the content of cytotoxic T cells in the naked mole rat is reduced, which correlates with the delayed thymus involution [27]. In the blind mole rat, thymus involution during aging proceeds normally, but the level of expression of T cell differentiation factors, as well as genes responsible for cytotoxicity, does not increase, which is typical for the rapidly aging rodents. In addition, analysis of immunoglobulin M in the blind mole rat indicates a shorter memory of B-cells [28]. These findings suggest that the properties of immune system could contribute to various strategies for extending life in the long-lived rodents. Perhaps some of these strategies involve canceling (like in the naked mole rat) or slowing down (like in the blind mole rat) the phenoptosis program.

**Figure 1:**
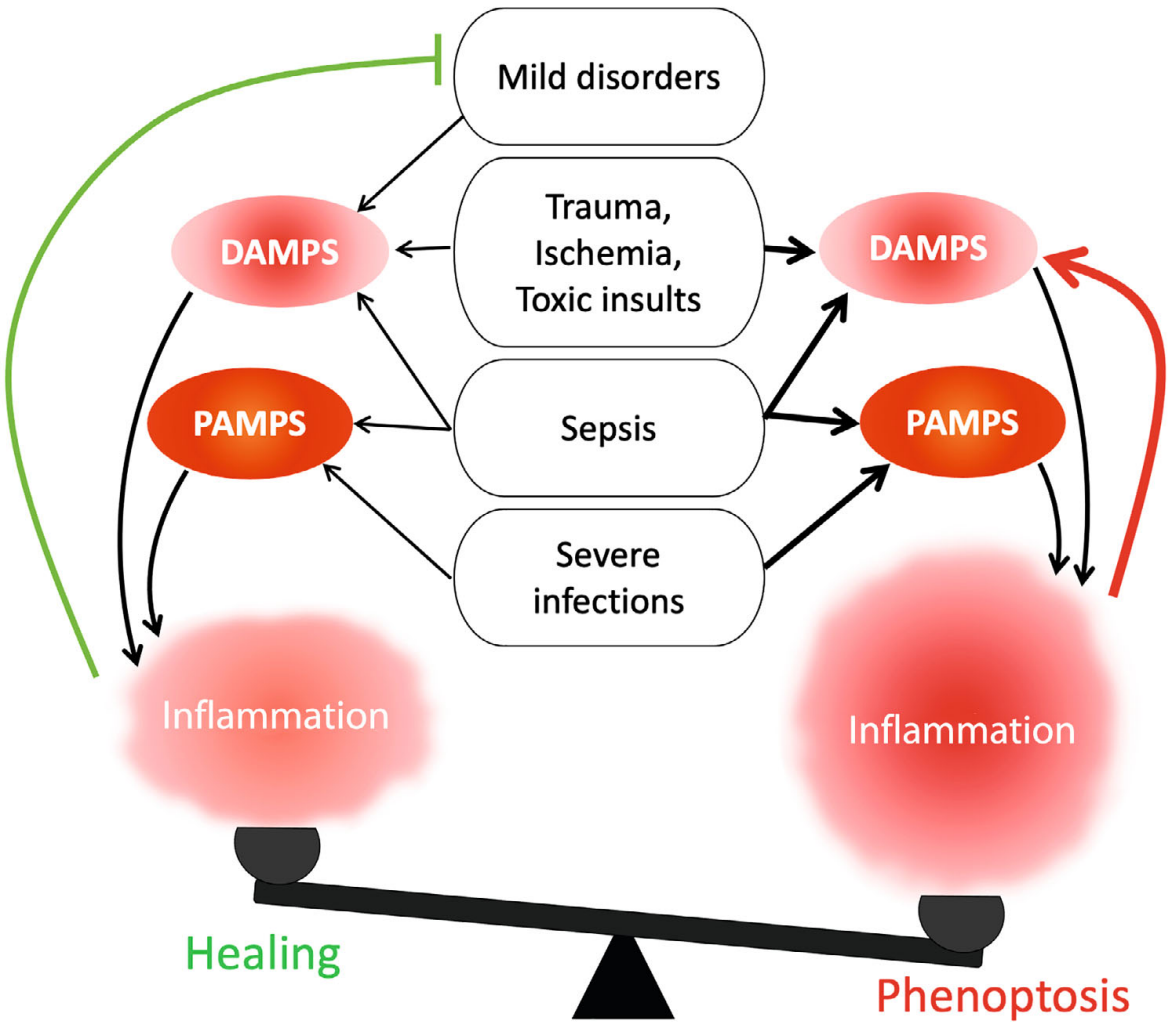
Scheme illustrating the hypothesis about the relationship between innate immunity and phenoptosis. By “*mild disorders*” diseases and injuries are meant in which the protective function of innate immunity prevails over the suicidal one. Sepsis, which develops as a reaction of the body to infection, is separated from the infections themselves in order to emphasize its non-contagiousness

The hypothesis proposed by us is, at first glance, deeply pessimistic, since it assumes that one of the body’s main defense systems is programmed to destroy the organism under critical conditions. However, if this hypothesis is correct and we know the mechanism underlying many severe diseases and aging, then there is a hope to develop drugs that would counteract it. After all, a modern human being can afford to suppress innate immunity at least temporarily, because we also have adaptive immunity, and access to antibiotics and antiviral drugs. The mitochondria-targeted antioxidants such as SkQ1 may become one of the prototypes of a “drug against phenoptosis”. We would like to believe that the use of such drugs can make our lives longer and less burdened by a wide variety of diseases (figure).

## Abbreviations

DAMPs: damage-associated molecular patterns
mitoROS: mitochondrial reactive oxygen species
NET: neutrophil extracellular traps
ROS: reactive oxygen species
